# (5*R*)-5-[(1*R*)-2,2-Dichloro-1-methyl­cyclo­prop­yl]-2-methyl­cyclo­hex-2-en-1-one

**DOI:** 10.1107/S1600536811021933

**Published:** 2011-06-11

**Authors:** Brahim Boualy, Mohamed Anoir Harrad, Larbi El Firdoussi, Mustapha Ait Ali, Corrado Rizzoli

**Affiliations:** a Laboratoire de Chimie de Coordination, Université Cadi Ayyad, Faculté des Sciences-Semlalia, BP 2390, 40001 Marrakech, Morocco; b Dipartimento di Chimica Generale ed Inorganica, Chimica Analitica, Chimica Fisica, Universitá degli Studi di Parma, Viale G. P. Usberti 17/A, I-43124 Parma, Italy

## Abstract

The title compound, C_11_H_14_Cl_2_O, was synthesized by the reaction of a dichloro­methane solution of (*R*)-carvone and potassium *tert*-butano­late in the presence of a catalytic amount of benzyl­triethyl­ammonium chloride in chloro­form. The cyclo­hexene ring adopts a half-boat conformation. The cyclo­propyl ring is unsymmetrical, the shortest C—C bond being distal to the alkyl-substituted C atom. The crystal packing is stabilized only by van der Waals inter­actions.

## Related literature

For background to and applications of dichloro­cyclo­propane derivatives, see: Hirota *et al.* (1996 ▶); Künzer *et al.* (1996 ▶); Ziyat *et al.* (2004 ▶); Fedorynski (2003 ▶). For the synthesis and structures of optically active dihalogenocylopropanes reported by our group, see: Ziyat *et al.* (2002 ▶); Boualy *et al.* (2009 ▶); Ziyat *et al.* (2006 ▶). For puckering parameters, see: Cremer & Pople (1975 ▶).
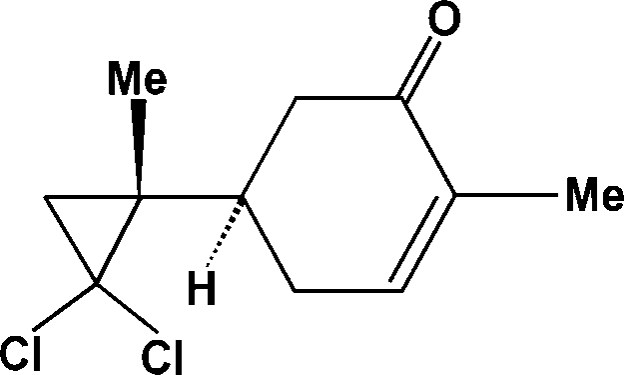


## Experimental

### 

#### Crystal data

C_11_H_14_Cl_2_O
*M* *_r_* = 233.12
Monoclinic, 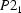

*a* = 6.5722 (3) Å
*b* = 8.4802 (4) Å
*c* = 10.8022 (5) Å
β = 104.435 (4)°
*V* = 583.04 (5) Å^3^
*Z* = 2
Cu *K*α radiation
μ = 4.73 mm^−1^
*T* = 294 K
0.25 × 0.20 × 0.14 mm

#### Data collection

Siemens AED diffractometer
Absorption correction: refined from Δ*F* (*DIFABS*; Walker & Stuart, 1983 ▶) *T*_min_ = 0.327, *T*_max_ = 0.519
2295 measured reflections
1576 independent reflections
1554 reflections with *I* > 2σ(*I*)
*R*_int_ = 0.057
3 standard reflections every 100 reflections  intensity decay: 0.3%

#### Refinement

*R*[*F* ^2^ > 2σ(*F* ^2^)] = 0.046
*wR*(*F* ^2^) = 0.126
*S* = 1.09
1576 reflections
130 parameters
1 restraint
H-atom parameters constrained
Δρ_max_ = 0.43 e Å^−3^
Δρ_min_ = −0.30 e Å^−3^
Absolute structure: Flack (1983 ▶), 394 Friedel pairs
Flack parameter: 0.00 (2)

### 

Data collection: *AED* (Belletti *et al.*, 1993 ▶); cell refinement: *AED*; data reduction: *AED*; program(s) used to solve structure: *SIR97* (Altomare *et al.*, 1999 ▶); program(s) used to refine structure: *SHELXL97* (Sheldrick, 2008 ▶); molecular graphics: *ORTEP-3 for Windows* (Farrugia, 1997 ▶) and *SCHAKAL97* (Keller, 1997 ▶); software used to prepare material for publication: *SHELXL97* and *PARST95* (Nardelli, 1995 ▶).

## Supplementary Material

Crystal structure: contains datablock(s) global, I. DOI: 10.1107/S1600536811021933/gk2382sup1.cif

Structure factors: contains datablock(s) I. DOI: 10.1107/S1600536811021933/gk2382Isup2.hkl

Supplementary material file. DOI: 10.1107/S1600536811021933/gk2382Isup3.cml

Additional supplementary materials:  crystallographic information; 3D view; checkCIF report

## Figures and Tables

**Fig. 1.**
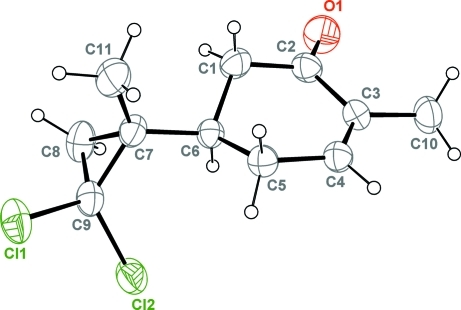
The molecular structure of the title compound, with displacement ellipsoids drawn at the 40% probability level.

**Fig. 2.**
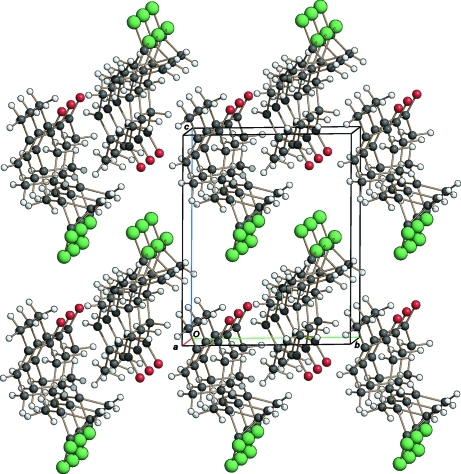
Crystal packing of the title compound approximately viewed along the a axis.

## supplementary crystallographic information

### Comment

Dichlorocyclopropanes play an important role in organic synthesis, due to the widespread occurrence of these structures in biologically active compounds (Hirota *et al.*, 1996; Künzer *et al.*, 1996). These compounds have found wide applications as substrates for the synthesis of many class of compounds such as pyrethroides (Ziyat *et al.*, 2004), benzocyclopropenes or cyclopentadiene derivatives (Fedorynski, 2003), which are not easily obtained using other starting materials. As a part of our ongoing research aimed at the synthesis of optically active dihalogenocylopropanes from terpenes (Ziyat *et al.*, 2002; Ziyat *et al.*, 2004; Boualy *et al.*, 2009; Ziyat *et al.*, 2006), the title compound has been prepared and its crystal structure is reported herein.

In the title compound (Fig. 1), the cyclohexene ring adopts a half-boat conformation [puckering parameters for ring C6/C1/C2/C3/C4/C5: *Q* = 0.478 (4) Å, θ = 126.7 (4)°, φ = -171.0 (6)°; Cremer & Pople, 1975] with atom C6 displaced by 0.645 (3) Å from the mean plane through the C1–C5 atoms. The cyclopropyl ring (C7–C9) is tilted by 57.64 (19)° with respect to this plane. As already observed in related compounds (Ziyat *et al.*, 2002), the geometry of the cyclopropyl ring is unsymmetrical, the distal bond to the alkyl-substituted C7 carbon atom (C8—C9 = 1.477 (6) Å) being significantly shorter than the vicinal bonds (C7—C8 = 1.518 (6) Å; C7—C9 = 1.500 (4) Å). The crystal packing (Fig. 2) is governed only by van der Waals interactions. The shortest intermolecular halogen···halogen separation is Cl1···Cl2^i^ = 3.990 (3) Å (symmetry code: (i) 2 - *x*, -1/2 + *y*, 1 - *z*).

### Experimental

Potassium *tert*-butanolate (4.00 g, 19.8 mmol) was added to (*R*)-carvone (1.47 g, 9.8 mmol) and benzyltriethylammonium chloride (0.02 g, 0.1 mmol) in dichloromethane (60 ml). The mixture was stirred for 10 min, then chloroform (0.8 ml, 9.8 mmol) was added dropwise over a period of 30 min. The mixture was stirred for 8 h at 25°C, and then hydrolyzed by addition of water (20 ml). The organic layer was separated and the aqueous layer was extracted with dichloromethane (3 × 10 ml). The combined organic extracts were dried over Na_2_SO_4_ and the solvent was removed under reduced pressure. Column chromatography on silica gel (hexane/ethyl acetate, 5:1 *v*/*v*) of the residue gave 1.24 g (5.3 mmol, 54% yield) of the title compound as colourless crystals. Slow evaporation of a chloroform solution of the title compound at 25°C afforded crystals suitable for X-ray crystallographic analysis. *M*.p. 392 K. [α]^20^_D_ = +7.72° (*c* 1, chloroform). ^1^H NMR (300 MHz, CDCl_3_, δ_p.p.m._): 1.18 (*s*, 2H), 1.3 (*s*, 3H), 1.78 (*d*, *J* = 0.9 Hz, 3H), 2.1 (*m*, 1H), 2.3–2.5 (*m*, 4H), 6.77 (*m*, 1H). ^13^C NMR (75 MHz, CDCl_3_, δ_p.p.m._): 15.6 (CH_3_), 16.0 (CH_3_), 28.5 (CH_2_), 32.5 (CH_2_), 40.8 (C*^q^*), 41.4 (CH_2_), 41.8 (CH), 65.8 (C*^q^*), 135.5 (═ C*^q^*), 144.5 (═CH), 199 (C═O). MS (70 eV) m/*z* (%): 233 [*M*^+^].

### Refinement

All H atoms were fixed geometrically and treated as riding, with C–H = 0.93–0.98 Å, and with *U*_iso_(H) = 1.2 *U*_eq_(C) or 1.5 *U*_eq_(C) for methyl H atoms. The absolute configuration of the molecule was established by the known chirality of the (*R*)-carvone starting material and, in spite of the low Friedel pair coverage (40%), the value of the resulting Flack (1983) parameter was in accordance with this configuration. For the inverted structure, the Flack parameter refined to 0.71 (3), and the values of *R*[*F*^2^>2σ(*F*^2^)] and *wR*(*F*^2^) increased to 0.0583 and 0.1695, respectively.

### Crystal data

**Table:**

C_11_H_14_Cl_2_O	*F*(000) = 244
*M**_r_* = 233.12	*D*_x_ = 1.328 Mg m^−^^3^
Monoclinic, *P*2_1_	Cu *K*α radiation, λ = 1.54178 Å
Hall symbol: P 2yb	Cell parameters from 48 reflections
*a* = 6.5722 (3) Å	θ = 22.5–35.9°
*b* = 8.4802 (4) Å	µ = 4.73 mm^−^^1^
*c* = 10.8022 (5) Å	*T* = 294 K
β = 104.435 (4)°	Irregular block, colourless
*V* = 583.04 (5) Å^3^	0.25 × 0.20 × 0.14 mm
*Z* = 2	

### Data collection

**Table:**

Siemens AED diffractometer	1554 reflections with *I* > 2σ(*I*)
Radiation source: fine-focus sealed tube	*R*_int_ = 0.057
graphite	θ_max_ = 69.8°, θ_min_ = 4.2°
θ/2θ scans	*h* = −7→6
Absorption correction: part of the refinement model (Δ*F*) (*DIFABS*; Walker & Stuart, 1983)	*k* = −10→6
*T*_min_ = 0.327, *T*_max_ = 0.519	*l* = −12→13
2295 measured reflections	3 standard reflections every 100 reflections
1576 independent reflections	intensity decay: 0.3%

### Refinement

**Table:**

Refinement on *F*^2^	Hydrogen site location: inferred from neighbouring sites
Least-squares matrix: full	H-atom parameters constrained
*R*[*F*^2^ > 2σ(*F*^2^)] = 0.046	*w* = 1/[σ^2^(*F*_o_^2^) + (0.083*P*)^2^ + 0.0912*P*] where *P* = (*F*_o_^2^ + 2*F*_c_^2^)/3
*wR*(*F*^2^) = 0.126	(Δ/σ)_max_ < 0.001
*S* = 1.09	Δρ_max_ = 0.43 e Å^−^^3^
1576 reflections	Δρ_min_ = −0.30 e Å^−^^3^
130 parameters	Extinction correction: *SHELXL*
1 restraint	Extinction coefficient: 0.014 (3)
Primary atom site location: structure-invariant direct methods	Absolute structure: Flack (1983), 394 Friedel pairs
Secondary atom site location: difference Fourier map	Flack parameter: 0.00 (2)

### Special details

**Table:**

Refinement. Refinement of *F*^2^ against ALL reflections. The weighted *R*-factor *wR* and goodness of fit *S* are based on *F*^2^, conventional *R*-factors *R* are based on *F*, with *F* set to zero for negative *F*^2^. The threshold expression of *F*^2^ > σ(*F*^2^) is used only for calculating *R*-factors(gt) *etc*. and is not relevant to the choice of reflections for refinement. *R*-factors based on *F*^2^ are statistically about twice as large as those based on *F*, and *R*- factors based on ALL data will be even larger.

### Fractional atomic coordinates and isotropic or equivalent isotropic displacement parameters (Å^2^)

**Table:**

	*x*	*y*	*z*	*U*_iso_*/*U*_eq_	
Cl1	1.34023 (13)	0.77757 (15)	0.58246 (8)	0.0824 (4)	
Cl2	0.90330 (12)	0.84462 (16)	0.47313 (8)	0.0858 (4)	
O1	0.7651 (5)	0.7920 (6)	−0.1253 (3)	0.0987 (12)	
C1	1.0031 (5)	0.7536 (6)	0.0774 (3)	0.0693 (10)	
H1A	1.0523	0.8597	0.0677	0.083*	
H1B	1.1099	0.6805	0.0652	0.083*	
C2	0.8020 (5)	0.7244 (5)	−0.0244 (3)	0.0636 (8)	
C3	0.6586 (5)	0.6053 (4)	0.0053 (3)	0.0554 (7)	
C4	0.7023 (5)	0.5357 (4)	0.1185 (3)	0.0571 (7)	
H4	0.6091	0.4590	0.1322	0.069*	
C5	0.8894 (4)	0.5702 (4)	0.2264 (3)	0.0548 (7)	
H5A	0.9982	0.4927	0.2270	0.066*	
H5B	0.8499	0.5627	0.3069	0.066*	
C6	0.9740 (4)	0.7342 (4)	0.2127 (3)	0.0496 (6)	
H6	0.8668	0.8101	0.2227	0.059*	
C7	1.1744 (4)	0.7717 (5)	0.3144 (3)	0.0584 (7)	
C8	1.2112 (6)	0.9436 (6)	0.3526 (4)	0.0799 (11)	
H8A	1.3547	0.9822	0.3731	0.096*	
H8B	1.1062	1.0197	0.3108	0.096*	
C9	1.1528 (4)	0.8301 (5)	0.4415 (3)	0.0614 (7)	
C10	0.4642 (6)	0.5669 (7)	−0.0993 (4)	0.0791 (11)	
H10A	0.3966	0.6630	−0.1344	0.119*	
H10B	0.3692	0.5055	−0.0644	0.119*	
H10C	0.5037	0.5079	−0.1655	0.119*	
C11	1.3618 (5)	0.6704 (8)	0.3127 (4)	0.0909 (15)	
H11A	1.3816	0.6673	0.2276	0.136*	
H11B	1.3386	0.5654	0.3395	0.136*	
H11C	1.4848	0.7138	0.3699	0.136*	

### Atomic displacement parameters (Å^2^)

**Table:**

	*U*^11^	*U*^22^	*U*^33^	*U*^12^	*U*^13^	*U*^23^
Cl1	0.0728 (5)	0.0887 (7)	0.0691 (5)	0.0062 (4)	−0.0133 (3)	−0.0101 (5)
Cl2	0.0610 (4)	0.1189 (9)	0.0741 (5)	0.0072 (5)	0.0107 (3)	−0.0351 (6)
O1	0.115 (2)	0.115 (3)	0.0616 (14)	−0.024 (2)	0.0147 (13)	0.028 (2)
C1	0.0660 (16)	0.081 (3)	0.0632 (17)	−0.0162 (17)	0.0201 (13)	0.007 (2)
C2	0.0756 (17)	0.066 (2)	0.0503 (15)	−0.0062 (16)	0.0182 (13)	0.0030 (17)
C3	0.0608 (14)	0.0537 (16)	0.0500 (14)	−0.0017 (14)	0.0104 (11)	−0.0062 (15)
C4	0.0616 (15)	0.0554 (17)	0.0544 (16)	−0.0067 (13)	0.0146 (12)	−0.0029 (15)
C5	0.0571 (14)	0.0535 (17)	0.0515 (15)	0.0014 (12)	0.0091 (11)	0.0047 (14)
C6	0.0467 (12)	0.0490 (16)	0.0512 (14)	0.0010 (10)	0.0087 (10)	−0.0009 (13)
C7	0.0443 (12)	0.0613 (18)	0.0666 (17)	−0.0028 (13)	0.0083 (11)	−0.0043 (17)
C8	0.075 (2)	0.064 (2)	0.090 (3)	−0.0173 (18)	0.0005 (17)	−0.001 (2)
C9	0.0511 (13)	0.0621 (19)	0.0632 (15)	0.0017 (13)	−0.0003 (11)	−0.0075 (17)
C10	0.081 (2)	0.086 (3)	0.0612 (18)	−0.019 (2)	0.0007 (15)	−0.007 (2)
C11	0.0462 (15)	0.124 (4)	0.097 (3)	0.015 (2)	0.0073 (16)	−0.027 (3)

### Geometric parameters (Å, °)

**Table:**

Cl1—C9	1.760 (3)	C6—C7	1.523 (4)
Cl2—C9	1.761 (3)	C6—H6	0.9800
O1—C2	1.202 (4)	C7—C9	1.500 (4)
C1—C2	1.514 (4)	C7—C11	1.506 (5)
C1—C6	1.529 (4)	C7—C8	1.518 (6)
C1—H1A	0.9700	C8—C9	1.477 (6)
C1—H1B	0.9700	C8—H8A	0.9700
C2—C3	1.470 (5)	C8—H8B	0.9700
C3—C4	1.323 (4)	C10—H10A	0.9600
C3—C10	1.515 (4)	C10—H10B	0.9600
C4—C5	1.497 (4)	C10—H10C	0.9600
C4—H4	0.9300	C11—H11A	0.9600
C5—C6	1.519 (5)	C11—H11B	0.9600
C5—H5A	0.9700	C11—H11C	0.9600
C5—H5B	0.9700		
			
C2—C1—C6	112.4 (2)	C11—C7—C8	118.4 (3)
C2—C1—H1A	109.1	C9—C7—C6	117.9 (2)
C6—C1—H1A	109.1	C11—C7—C6	115.8 (3)
C2—C1—H1B	109.1	C8—C7—C6	116.5 (3)
C6—C1—H1B	109.1	C9—C8—C7	60.1 (3)
H1A—C1—H1B	107.8	C9—C8—H8A	117.8
O1—C2—C3	122.0 (3)	C7—C8—H8A	117.8
O1—C2—C1	121.5 (3)	C9—C8—H8B	117.8
C3—C2—C1	116.5 (3)	C7—C8—H8B	117.8
C4—C3—C2	120.3 (3)	H8A—C8—H8B	114.9
C4—C3—C10	122.8 (3)	C8—C9—C7	61.3 (3)
C2—C3—C10	116.9 (3)	C8—C9—Cl1	119.2 (2)
C3—C4—C5	125.4 (3)	C7—C9—Cl1	120.2 (2)
C3—C4—H4	117.3	C8—C9—Cl2	119.1 (3)
C5—C4—H4	117.3	C7—C9—Cl2	120.4 (2)
C4—C5—C6	110.7 (3)	Cl1—C9—Cl2	109.55 (18)
C4—C5—H5A	109.5	C3—C10—H10A	109.5
C6—C5—H5A	109.5	C3—C10—H10B	109.5
C4—C5—H5B	109.5	H10A—C10—H10B	109.5
C6—C5—H5B	109.5	C3—C10—H10C	109.5
H5A—C5—H5B	108.1	H10A—C10—H10C	109.5
C5—C6—C7	113.1 (3)	H10B—C10—H10C	109.5
C5—C6—C1	109.1 (3)	C7—C11—H11A	109.5
C7—C6—C1	112.1 (2)	C7—C11—H11B	109.5
C5—C6—H6	107.4	H11A—C11—H11B	109.5
C7—C6—H6	107.4	C7—C11—H11C	109.5
C1—C6—H6	107.4	H11A—C11—H11C	109.5
C9—C7—C11	117.6 (3)	H11B—C11—H11C	109.5
C9—C7—C8	58.6 (3)		
